# Influence of Manually Adjustable Photovoltaic Array on the Performance of Water Pumping Systems

**DOI:** 10.1002/gch2.201900009

**Published:** 2019-08-07

**Authors:** Vinamrita Singh

**Affiliations:** ^1^ Department of Applied Sciences and Humanities Ambedkar Institute of Advanced Communication Technologies & Research Geeta Colony Delhi 110031 India

**Keywords:** photovoltaics, solar energy, solar water pumps, tilt angle

## Abstract

This work presents a theoretical procedure to calculate various parameters needed for the installation of a photovoltaic water pump (PVWP) system. Specifically, the monthly optimum tilt angle of PV arrays is calculated, which will allow larger quantities of water to be pumped each month. For this, a simple performance optimization procedure is developed using solar radiation data. The investigations are based on the solar radiation data of New Delhi, India. The total amount of water pumped is compared with that by keeping the solar panels fixed throughout the year. The total amount of water that can be extracted by using manually adjustable arrays is found to be greater by 3–5%. This may be an alternative to an automatic sun tracker, which is not only expensive but also requires more space and maintenance. This method allows for analyzing the performance of low‐cost water pumping systems that may be used for both domestic and irrigation purposes.

## 1 Introduction

The increasing energy demand of the world constantly requires new technology, which is based on renewable energy resources. Out of the various renewable energy resources available, solar energy is the most promising as the sun gives out an enormous amount of heat and light. Many solar energy based applications are available commercially, such as solar cells, solar heaters, concentrators, distillers, etc.^1^, ^2^, ^3^, ^4^ These devices have further been integrated with various appliances to achieve clean energy driven technology. The photovoltaic powered water pump is one such application, which has received great attention in the past decades.^5^, ^6^, ^7^ Water pumping is heavily required for many purposes throughout the world, for which the conventional electricity and diesel driven water pumps are dominantly used. However, electricity and diesel based water pumps have several drawbacks.^8^, ^9^, ^10^ In rural areas, where grid power is not accessible, or power cuts are regular, traditional electricity based water pump poses a limitation. Diesel based water pumps require fuel to be transported to the location, are noisy and polluting, and have significant maintenance requirements.^8^ Altogether, these pumping systems are not cost effective.

Photovoltaic water pumping (PVWP) systems are gaining popularity both in the developed and developing countries. The developed countries aim to reduce environmental pollution through the use of this technology, while the developing countries need PVWP as solar energy may be the only available energy in some underdeveloped places as well as remote locations.^11^ The use of PV based pumps is also location dependent, as the performance of photovoltaic devices is governed by several factors such as total incident radiation and temperature.^12^ The rapid decline in the PV module prices over the last five years has reduced the overall costs of PVWP system.^13^ Advancement in PVWP systems has resulted in its broad use from irrigation to domestic use. Furthermore, systems which are integrated either with batteries or storage tanks, make water accessible even when sunlight is not available. The initial high‐cost factor associated with installing PVWPs hinders its widespread use; however, these systems are very economical in the long run as compared to diesel or electricity powered pumps. Other advantages of PVWPs are their low environmental impact, does not require on‐site operator, are simple to operate, and sustain longer with easy maintenance.^8^ Moreover, modern well‐equipped systems come in a variety of designs and assemblies, providing a wide range of choices depending upon the user's requirements.

**Figure 1:**
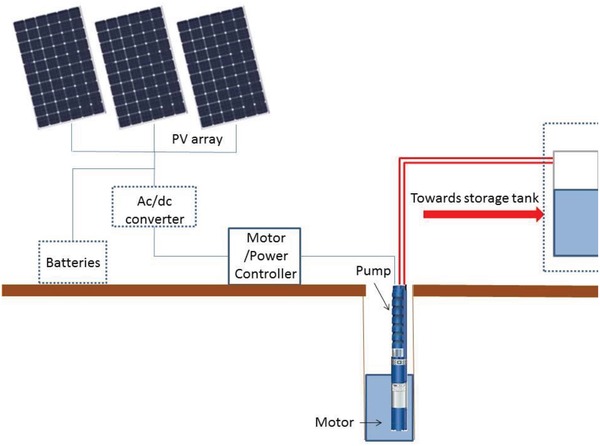
Schematic diagram of a solar water pump. Optional components are shown in dashed frames.

The basic components of a PVWP system, shown in **Figure** **1**, comprise PV modules, motor and pump. Other components may include ac/dc converter, batteries, and storage tank. The PV modules are chosen depending upon the power requirement; a number of solar cells are connected in series and parallel combinations in order to provide the necessary voltage and current. The performance of PV devices, and subsequently, the water pump, depends on the incident radiation. Fixed PV panels are not able to fully utilize the solar radiation throughout the day. Therefore, automatic sun trackers and maximum power point trackers (MPPT) may be used in order to increase the output.^14^ However, these significantly add to the cost of PVWP system. The PV array is connected to a motor either directly in case of dc motors, or through an inverter (dc to ac converter) if an ac motor is used. Direct coupling mode reduces the cost of the system. The most cost‐effective installation is the one which has a water storage tank. Excess water can be stored for use at night or when the solar radiation is not sufficient to run the pump. A more expensive alternative to water storage is to connect the PV arrays with batteries. During times when PV cells are not working, the batteries provide the electricity to run the motor. Although this option is both expensive and requires maintenance, it is more regulated in terms of the system output.^15^

One of the major challenges in installing PVWPs is that every site requires different parameters. The reasons attributed to this are that solar radiation, climate conditions, available space, and individual requirements differ in every case. Therefore, case studies have to be performed every time PVWP has to be installed. Moreover, the preferences regarding the configuration also change the output delivered by the system. This makes it difficult to model the PVWP yield theoretically. Many studies have been carried out on PVWP systems in different locations of the world. Research has been focused on various aspects of solar water pump functioning, designs, performance, degradation, etc.^5^ Carr and Pryor studied the efficiency and degradation of solar panels based on different materials.^16^ Chandel et al. demonstrated the long term effect of field exposure on water pumping system in the Himalayan region of India.^17^ Katan et al. studied the effect of using MPPT and sun trackers.^18^ Various researchers analyzed the effect of orientation of PV arrays on the output.^19^, ^20^, ^21^ The socioeconomical as well as environmental aspect has also been investigated, supporting the advantages of using PVWP systems.^22^, ^23^, ^24^, ^25^ Various techniques for increasing the efficiency of PVWPs has been highlighted in literature based on both experimental and theoretical simulations.^26^, ^27^, ^28^, ^29^, ^30^ Simple methods of reducing the PV module temperature can increase the amount of water pumped.^28^, ^31^ Studies based on using inverters, comparison, and compatibility between different types of pumps used has also been done, which gives a strong insight into the field of solar powered water systems.^15^

Theoretical studies have also been carried out to determine the performance of water pumping systems. Gad et al. developed a program to simulate the functioning of a PVWP.^32^ Several authors have simulated the water pumped in terms of various parameters such as insolation and individual components.^33^, ^34^, ^35^, ^36^ Due to the high variability and dependency of the PVWP performance on different parameters, modeling the behavior of these systems is critical and needs more attention.

In the present work, theoretical studies have been carried out to predict the water pumped through a PVWP system. A simple optimization procedure has been developed using solar radiation data. Optimum tilt angle has been calculated for each month of the year. The effect of manually varying the PV array angle on a monthly basis has been analyzed, and has been compared with the amount of water pumped by keeping the solar panels fixed throughout the year. The method is developed for direct‐coupled PV water pumps and exhibit the performance of low‐cost water pumping systems that may be used for both domestic and irrigation purposes.

## 2 Site Survey

**Figure 2:**
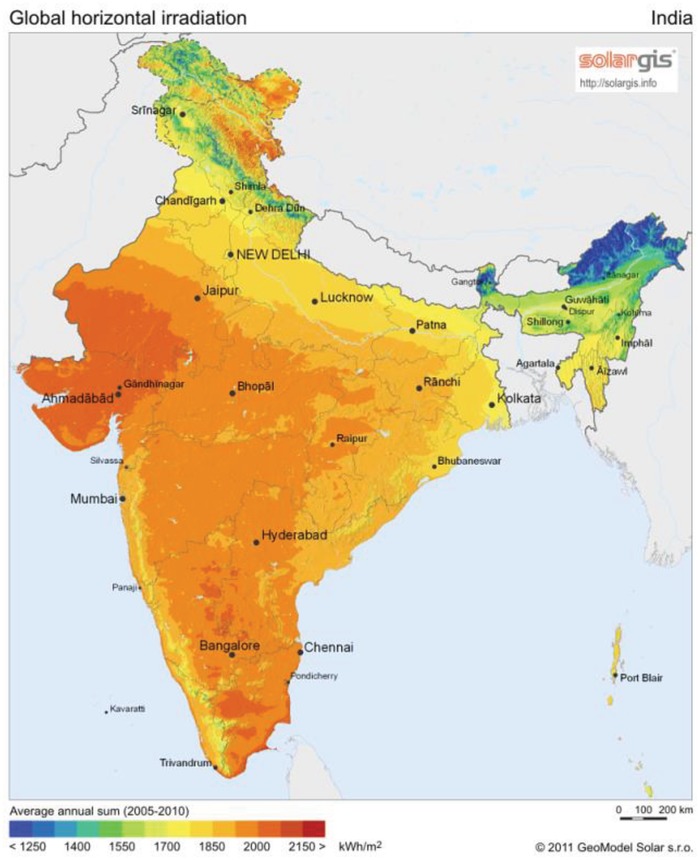
Average solar energy distribution in India.^39^

In the present work, the site under investigation is the capital city of India, i.e., Delhi. India receives a considerable amount of solar radiation throughout the country, thus making it a wise place to use solar‐powered appliances. The average solar energy distribution in India is shown in **Figure** **2**. India gets 5000 TWh of solar insolation every year, with most regions receiving 4–7 kWh m^−2^ per day.^37^ Delhi is one of the hottest regions in India, and receives an annual average of about 5.20 kWh m^−2^ per day energy from the sun.^38^ There is abundant use of groundwater for domestic and agricultural use in Delhi. Considering the large water demand as well as adequate solar energy available in Delhi, the use of PV panels to power water pumping systems is a justifiable choice.

**Figure 3:**
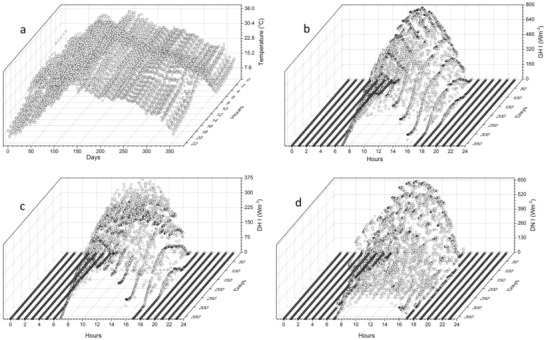
a) Ambient temperature, b) total solar radiation, c) direct, and d) diffused components of solar radiation for each hour and day of the year.

Since the efficiency of PV arrays depends on the incident solar radiation and temperature, data taken at regular intervals is essential for accurate configuration of PVWP systems. The present work is based on the hourly data given by the National Renewable Energy Laboratory, USA.^40^ **Figure** **3**a–d shows the ambient temperature, total solar radiation, direct and diffuse components of solar radiation, respectively, for one year. The total solar radiation is usually given for a horizontal surface. Therefore, the direct and diffused components are required in order to calculate the total incident radiation at any tilted surface. It can be seen from the figures that the maximum temperature and incident radiation are received for the months of March to May and September and October. There is a significant drop in both parameters during July and August.

## 3 Theoretical Background

Static PV arrays are mounted facing south with a constant slope that is equal to the site's latitude. Although such installations are convenient in terms of system design and cost, they result in significant power loss. On the other hand, the use of automatic tracking system increases power output, but makes the unit complex and unreliable as well, especially for domestic use. In order to average out the advantages and disadvantages of the above two systems, a manual tracking system can be constructed in such a way that it needs to be adjusted only a few times during the year. Such a system gives higher output as compared to the yearly constant slope system, does not add appreciable cost, and can be handled by untrained domestic users. The formulas below describe the methods utilized for calculating an optimal monthly slope of the PV array. The method can easily be extended for finding optimum slopes which can be manually adjusted quarterly, every 15 days, daily, etc.

### 3.1 Total Radiation on Tilted Surface

The total radiation on a tilted surface is given by^41^

(1) $$H_{\text{t}}=R_{\text{B}}\;H_{\text{B}}+R_{\text{D}}H_{\text{D}}$$

where *H*_B_ and *H*_D_ are direct and diffused solar radiation components on a horizontal plane; *R*_B_ and *R*_D_ are direct and diffused radiation tilt factors, given by

(2) $$R_{\text{B}}=\frac{\cos\left(L-\alpha \right)\cos\left(\delta \right)\cos\left(h\right)+\sin\left(L-\alpha \right)\text{sin}\left(\delta \right)}{\cos\left(L\right)\cos\left(\delta \right)\cos\left(h\right)+\sin\left(L\right)\text{sin}\left(\delta \right)}$$

(3) $$R_{\text{D}}=\frac{1+\text{cos}\alpha }{2}$$

Here, *L* is the latitude of site; α is the tilt angle of the PV array; δ is the solar declination angle; *h* is solar hour angle. The declination and solar hour angle can be calculated using

(4) $$\delta \;=\;23.5\text{sin}\left[\frac{360}{365}\left(N+284\right)\right]$$

(5) $$h\;=\left(12-t\right)\;\times \;15^{{}^{\circ}}$$

where *N* is the number of the days from January 1st and *t* is the time in hours.

### 3.2 Photovoltaic Array Characteristics

The current–voltage characteristic of a solar cell is given by

(6) $$I\;=I_{L}\;-I_{0}\left[{\text{e}}^{\left(\frac{qV}{kT}\right)}-1\right]$$

where *I*_L_ is the photocurrent *I*_0_ is the reverse saturation current, and *T* is the cell temperature in Kelvin. The open circuit voltage (*V*_OC_) and short circuit current (*I*_SC_) are important parameters which determine the efficiency of solar cells, and can be determined by setting current and voltage to zero, respectively, in the above equation. The photocurrent of a solar cell depends upon the illumination intensity according to

(7) $$I_{L}\;\left(H\right)=\left(\frac{H}{H_{\text{S}}}\right)\;\;I_{\text{L}}\left(H_{\text{S}}\right)$$

where the subscript “s” denotes the standard conditions of 1000 Wm^−2^ and 1.5 air mass ratio at 25 °C. The output power of a photovoltaic cell is calculated by *P* = FIV; where *F* is the fill factor. The maximum power deliverable by a solar cell is *P*_m_ = *I*_m_ *V*_m_.

### 3.3 Pump Characteristics

The power consumption of a pump is given by

(8) $$P_{\text{P}}=\;nAP\;=\frac{\rho gH_{\text{h}}}{\eta_{\text{p}}}$$

where *n* is the number of cells per meter sq. of PV array; *A* is the array area; ρ is the density; *g* is the acceleration due to gravity; *H*_h_ is the total head; η_p_ is the pump overall efficiency. The water flow rate can be found as

(9) $$q\;=\frac{\eta_{\text{p}}nP_{\text{m}}}{\rho gH_{\text{h}}}$$

The above equations can be used to assemble an efficient solar water pump system. The detailed calculations and parameters used will be elaborated in Section 4.

## 4 Results and Discussion

**Figure 4:**
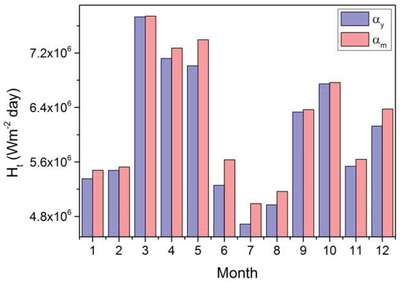
Daily incident total solar radiation for a yearly constant slope (*α_y_*) and monthly varying slope (α_m_).

Fixed PV array systems are installed facing south with an optimal yearly angle equal to the site's latitude. The latitude of Delhi is *L* = 28.61°. In order to calculate the daily incident total solar radiation, *H*_t_, for a fixed slope of *α_y_* = *L*, Equations (1)–(5) were used. The values of *R*_B_ and *R*_D_ were calculated for different values of *h* and δ from which *H*_t_ was obtained. The daily incident total solar radiation for a yearly constant slope is shown in **Figure** **4**. Figure 4 also shows the *H*_t_ when the PV array angle is adjusted every month. There is a considerable difference between the *H*_t_ values corresponding to α_y_ and α_m_, especially from April to August. *H*_t_ is higher when the PV array slope is varied monthly. This will increase the performance of the water pump with an insignificant increase in system cost, and it can be readily adjusted by nontechnical users. The difference is quite important considering that the quantity of water pumped depends on the amount of solar radiation that falls on PV array. The surplus water can be stored in water tanks for use in times when the pump system is not operating.

**Figure 5:**
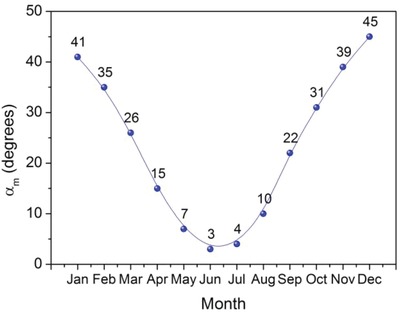
Theoretically calculated values of monthly optimum slopes.

The following approach has been used to calculate the optimal monthly slope and hence the *H*_t_. The available hourly data was summed to obtain the day's total radiation. This was further added and divided by the number of days to find the monthly average radiation in terms of direct and diffused components. The values of *R*_B_ and *R*_D_ were calculated for α = 0°–90° with a step size of 1°. The values thus obtained were put in Equation (1) to determine *H*_t_ corresponding to different αs. The monthly optimum slope then corresponds to the angle at which *H*_t_ is maximum. The procedure is repeated for each month of the year. The theoretically calculated values of α_m_ are shown in **Figure** **5** for panels facing in the south direction.

**Figure 6:**
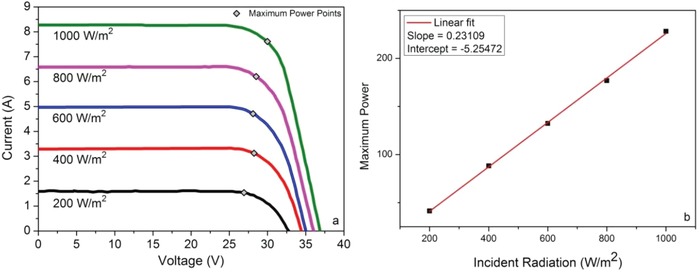
a) *IV‐*characteristics of a solar panel at different light intensities, b) straight line fit of maximum power point versus illumination intensity.

After estimating the optimal monthly slope, it is required to model the output of both the PV arrays and the water pump in order to determine how much water can be pumped each month. The same calculations can be made using a yearly constant slope for comparative analysis. **Figure** **6**a shows the *IV*‐characteristics of a multicrystalline solar module, which has 60 cells connected in series and parallel combinations. Each cell area is 243.36 cm^2^. The *IV*‐characteristics depend on the illumination intensity as can be seen from the figure. As mentioned earlier, the important solar cells parameters are open circuit voltage, short circuit current and the fill factor. The values of these parameters at different illumination intensities are given in **Table** **1**.

**Table 1:** Solar cell parameters for different illumination intensities

	200 Wm^−2^	400 Wm^−2^	600 Wm^−2^	800 Wm^−2^	1000 Wm^−2^
*I*_SC_ [A]	1.59	3.29	4.97	6.58	8.27
*V*_OC_ [V]	32.84	34.42	35.01	36.05	36.86
*F* [%]	79.36	77.96	76.11	74.54	74.86

A PV array usually operates at a maximum power point (*P*_m_). The *P*_m_ for each curve is also shown in Figure 6a. The relation between different solar radiation intensity and the maximum power point can be deduced by the straight line curve fit as shown in Figure 6b. The following relation has been obtained for the current data

$$\;P_{\text{m}}=\;0.23109H-5.25472$$

The next step is to calculate the amount of water pumped per unit array area. For this, we make use of Equation (9). The quantity “*q*” can be averaged over the whole day to obtain the daily amount of water pumped. The calculations have been made for *n* = 60, *H*_h_ = 50 m, and η_p_ = 85%. The pump can operate only after the solar panels deliver a threshold current. Therefore, water flow has to be calculated by averaging over only the active hours, i.e., during the time the solar panels are able to run the pump. The active hours will differ throughout the year, and has to be taken into consideration. **Table** **2** gives the average values of the time the pump operates each month based on the minimum incident solar radiation per unit area of the PV array and the average incident radiation. The calculations have been performed for three different incident radiations, i.e., for more than 300, 400, and 500 Wm^−2^. Here, the data has been corrected using monthly tilt angles throughout the calculations, and the active hours have been calculated on the basis of time during which the total incident radiation was greater than or equal to 300, 400 and 500 Wm^−2^.

**Table 2:** Active hours of the water pump along with average incident radiation

	*H*_t_ yearly						*H*_t_ monthly					
	300 Wm^−2^		400 Wm^−2^		500 Wm^−2^		300 Wm^−2^		400 Wm^−2^		500 Wm^−2^	
	Δ*t* ^a)^	*H* ^a)^	Δ*t*	*H*	Δ*t*	*H*	Δ*t*	*H*	Δ*t*	*H*	Δ*t*	*H*
Jan	7	5.18	7	5.18	6	5.18	7	5.32	7	5.32	7	5.32
Feb	9	7.02	9	7.02	9	7.02	9	7.11	9	7.11	9	7.11
Mar	9	7.61	9	7.61	9	7.61	9	7.34	9	7.34	9	7.34
Apr	10	7.36	9	7.04	9	7.04	10	7.61	9	7.25	9	7.25
May	10	6.62	9	6.32	8	5.89	10	7.18	9	6.81	8	6.32
Jun	9	5.15	7	4.43	7	4.43	9	5.62	8	5.23	7	4.82
Jul	9	4.35	7	3.72	4	2.34	9	4.69	7	4.01	5	3.03
Aug	9	4.62	7	3.94	6	3.49	9	4.84	7	4.12	6	3.64
Sep	10	6.82	9	6.44	8	5.96	10	6.85	9	6.47	8	5.99
Oct	9	6.86	8	6.54	8	6.54	9	6.87	8	6.56	8	6.56
Nov	8	6.12	8	6.12	7	5.63	8	6.28	8	6.28	8	6.28
Dec	7	6.02	7	6.02	7	6.02	7	6.33	7	6.33	7	6.33

^a)^ Unit of Δ*t* is hours and *H* is kWh m^−2^.

**Figure 7:**
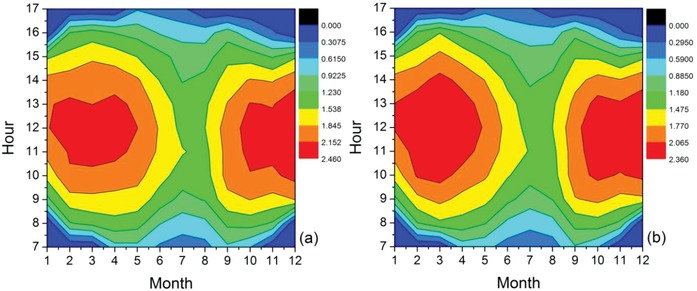
Amount of water pumped (in m^3^) during active hours every month for a) constant slope and b) for monthly varying slope.

The daily average amount of water pumped for different incident radiations during each month is given in **Table** **3**. The data is shown for both static inclination and monthly varying inclination at a minimum intensity of 300, 400, and 500 Wm^−2^. The maximum water is pumped during March–April for all incident radiations, while the minimum water is pumped during July. Thus, we see that the amount of water pumped not only depends on the solar radiation each month but is also dependent on the threshold operating radiation that we set to turn on the machine. A color graph for the amount of water pumped during active hours every month is shown in **Figure** **7**a,b.

**Table 3:** Average quantity of water pumped (in m^3^) per day at different incident intensities

	*H*_t_ yearly			*H*_t_ monthly		
	300 Wm^−2^	400 Wm^−2^	500 Wm^−2^	300 Wm^−2^	400 Wm^−2^	500 Wm^−2^
Jan	12.07	12.07	10.96	12.43	12.43	12.43
Feb	16.4	16.4	16.4	16.6	16.6	16.6
Mar	17.8	17.8	17.8	17.16	17.16	17.16
Apr	17.17	16.43	16.43	17.76	16.95	16.95
May	15.39	14.71	13.72	16.72	15.89	14.77
Jun	11.89	10.28	10.28	13.04	12.14	11.21
Jul	9.96	8.57	5.42	10.81	9.25	7.02
Aug	10.61	9.09	8.07	11.15	9.52	8.43
Sep	15.86	14.99	13.91	15.94	15.07	13.97
Oct	16.01	15.30	15.30	16.04	15.33	15.33
Nov	14.29	14.29	13.6	14.66	14.66	14.66
Dec	14.10	14.10	14.10	14.84	14.84	14.84
Total	5210.18	4981.12	4719.94	5381.27	5157.42	4959.28

In order to understand the importance of adjusting the PV arrays every month, we need to compare the total amount of water pumped with that by keeping a constant PV array slope. Increase in the amount of water pumped for 300, 400, and 500 Wm^−2^ are ≈171, 176, and 239 m^3^, respectively, which corresponds to a percentage increase of 3–5%. There is a reasonable difference in the amount of water obtained in the two cases. If we take the average water consumption of a person in urban India to be 135 L per day, even 171 m^3^ equates to an extra 171 000 L per year; this is sufficient for the need of more than 3.5 persons per day. So, a surplus of water can be extracted using the same configuration of solar panels without much labor and added cost. This highlights the importance of manual tracking system. Thus, it is essential to adjust the slope of the array in order to gain more value out of the cost invested in a solar water pump system.

The above calculations have been performed considering the specifications of any one type of PV array and pump. However, the method presented above can be used to analyze the outcome of any combination of solar panels and pumps with varying efficiencies. The water needs are especially different for different purposes, so accordingly, the number of solar cells, battery backup and/or water storage tank can be set up to meet the individual demand.

## 5 Conclusion

Solar‐energy‐based water pumps are environmentally friendly and cost‐effective in the long run. Therefore, attempts to understand its performance have been made in the present work. The investigations are based on the solar radiation data of New Delhi, India. An alternative to an automatic sun tracker has been presented, which only requires adjusting a solar panel monthly. The total amount of water that can be extracted by using a fixed array as well as the manually adjustable array has been compared, and it is found that the latter method increases the water quantity obtained by 3–5%. Thus, it is recommended that such provision be made available to the buyers so that they can get the maximum benefit of their product.

## Conflict of Interest

The author declares no conflict of interest.
